# Case in Which Death Was Caused by Eating Raw Rice

**Published:** 1847-07

**Authors:** Debert Howell


*Case in which death was caused by eating raw rice.* By Debert Howell, M. D.—Maria W——, a servant, aged twenty-two, previously in moderate health, but pale and anæmic, was taken suddenly ill with pain in the chest, while walking out in the evening of December 17th, 1846. At half-past seven, half an hour from the attack, she was suffering severe pain in the left hypochondriac region, attended by great restlessness. Percussion over the region of the stomach was not unusually loud. On inquiry, it proved that she had eaten in the afternoon, before her tea, a tumblerful of raw rice, mixed with milk, which she had been in the habit of eating, as well as arrow-root, sago, &c., in a raw state. The pain evidently arising from distention, caused by swelling of the rice in contact with the tea, and aided by the heat of the body, half a drachm of sulphate of zinc was administered as an emetic, which, failing to act, was repeated after twenty minutes. The stomach was then relieved, first of what appeared to be tea and wash, and afterwards, at intervals, of a large quantity of half-swollen rice, equal in bulk to an ordinary dinner-plate, piled ; and she felt considerable relief from pain. The stomach-pump was not employed in this case, because it did not appear calculated to relieve the stomach of its half solid contents ; in similar cases, however, it might prove useful by favoring the escape of gas. At eleven the following morning the pain increased suddenly, violently, with cold extremities, and feeble pulse, great abdominal tenderness, and she died at 4 P. M. On examination of the body, extensive peritonæal inflammation presented itself, with deposition of lymph agglutinating the intestines, and a copious effusion of turbid serum into the cavity of the abdomen. The stomach and duodenum were empty, with the exception of a few grains of raw rice at the pylorus, and perfectly free from inflammation. The small intestines were gorged throughout with a quantity of the same raw material that she had been in the habit of eating, apparently rice, arrow-root, &c., some raw and hard, and in parts so distending the intestine as to give the sensation to the fingers of feeling a bag of marbles, and and some in a half digested state. The large intestines were loaded with fæces. The heart was small, the lungs healthy. It is remarkable that the stomach was perfectly free from inflammation.—*London Lancet.*

